# Revisiting the Brain Renin-Angiotensin System—Focus on Novel Therapies

**DOI:** 10.1007/s11906-019-0937-8

**Published:** 2019-04-04

**Authors:** Liwei Ren, Xifeng Lu, A. H. Jan Danser

**Affiliations:** 1 Division of Pharmacology and Vascular Medicine, Department of Internal Medicine, Erasmus MC, Wytemaweg 80, 3015 CN Rotterdam, The Netherlands; 2 AstraZeneca-Shenzhen University Joint Institute of Nephrology, Department of Physiology, Shenzhen University Health Science Center, Shenzhen University, Shenzhen, China

**Keywords:** Brain renin-angiotensin system, Aminopeptidase A inhibitor, AT_2_ receptor, Angiotensinogen, Kidney, Sympathetic nervous system, Stroke

## Abstract

**Purpose of Review:**

Although an independent brain renin-angiotensin system is often assumed to exist, evidence for this concept is weak. Most importantly, renin is lacking in the brain, and both brain angiotensinogen and angiotensin (Ang) II levels are exceptionally low. In fact, brain Ang II levels may well represent uptake of circulating Ang II via Ang II type 1 (AT_1_) receptors.

**Recent Findings:**

Nevertheless, novel drugs are now aimed at the brain RAS, i.e., aminopeptidase A inhibitors should block Ang III formation from Ang II, and hence diminish AT_1_ receptor stimulation by Ang III, while AT_2_ and Mas receptor agonists are reported to induce neuroprotection after stroke. The endogenous agonists of these receptors and their origin remain unknown.

**Summary:**

This review addresses the questions whether independent angiotensin generation truly occurs in the brain, what its relationship with the kidney is, and how centrally acting RAS blockers/agonists might work.

## Introduction

Angiotensinogen is the precursor of all angiotensin (Ang) metabolites. Although its major source is the liver, additional sites of angiotensinogen synthesis have been reported, the most important of which are the brain, kidney, and adipose tissue [1•, 2–7]. Renin, in contrast, is derived from one source, the kidney. Its precursor, prorenin, like angiotensinogen, remarkably has several sources, including the kidney, ovaries, testis, and adrenal [8]. Yet, given the fact that prorenin is inactive, it would require a (local?) activation mechanism to be of importance. Here, the (pro)renin receptor, which binds and activates prorenin in vitro, has been proposed as a major player [9]. Unfortunately however, its affinity for prorenin is too low to allow this phenomenon to play any role in vivo [10], and the concept of (pro)renin receptor-prorenin interaction as a unit allowing local Ang I-generating activity is now being abandoned [11]. This does not mean that the (pro)renin receptor has no role at all—in contrast, given its ubiquitous abundance, its link with vacuolar H^+^-ATPase, and the lethal consequences of its deletion, it turns out to be of vital importance [12–15], yet apparently independently of the renin-angiotensin system (RAS). Taken together, the various sites of renin, prorenin, and angiotensinogen synthesis allow multiple possibilities for angiotensin generation, e.g., in circulating blood from renal renin and hepatic angiotensinogen, or at tissue sites, from either locally synthesized angiotensinogen and prorenin, or renin, prorenin, and angiotensinogen taken up from blood. Yet, regarding prorenin, we still lack a detailed insight into how it might display activity. This review focuses on the brain RAS, critically addressing the questions whether independent angiotensin generation occurs in the brain, what its relationship with the kidney is, and how centrally acting RAS blockers (in particular, the recently introduced aminopeptidase A inhibitors) and activators (Ang II type 2 (AT_2_) and Mas receptor agonists) might work.

## Independent Angiotensin Generation in the Brain?

**Fig. 1:**
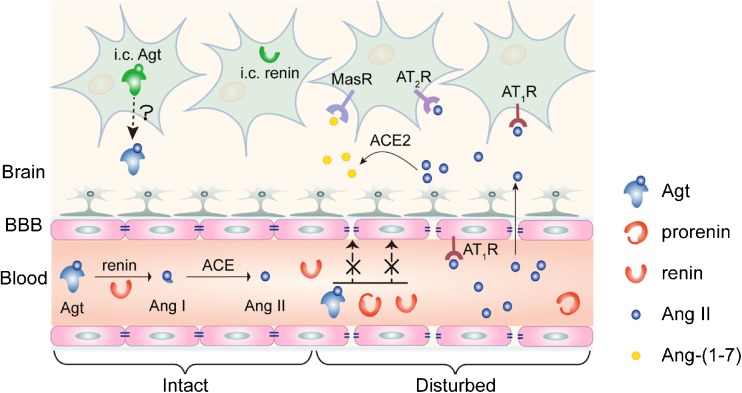
Current understanding of the origin of brain renin-angiotensin system (RAS) components. Circulating renin, prorenin, and angiotensinogen (Agt) are unlikely to pass the blood-brain barrier (BBB). Intracellular (i.c.) renin does not contribute to brain RAS activity. Agt has been shown in brain cells and cerebrospinal fluid, yet whether actual release from cells into cerebrospinal fluid occurs remains unclear, nor do we know how brain cell-derived Agt contributes to local angiotensin (Ang) generation in the absence of renin. Circulating Ang II may bind to brain Ang II type 1 or 2 (AT_1_, AT_2_) receptors (R) outside the BBB, or could diffuse into the brain under conditions where the BBB is disturbed (right part of the figure), like in hypertension. Possibly, such diffusion results in local formation of Ang-(1-7) and subsequent Mas receptor stimulation

Given the presence of the blood-brain barrier, diffusion of circulating renin, prorenin, or angiotensinogen into the brain is impossible (Fig. 1). Although early studies were able to demonstrate renin-like activity in the brain [16], its origin remained uncertain. Here, one has to consider that brain tissue, when homogenized, of course contains minute amounts of trapped blood, and thus brain renin measurements have to be corrected for such admixture. Recently, making use of different mouse brain nuclei, we performed such correction, and in parallel experiments perfused the brain with buffer to remove as much blood as possible prior to brain tissue homogenization [1•]. Data revealed that, although renin was easily detectable in brain nuclei, it could be entirely explained based on the presence of trapped blood in brain tissue—indeed, it disappeared after buffer perfusion. Moreover, we found no evidence for the synthesis of prorenin in the brain, not even under circumstances where activation of the brain RAS has been proposed, i.e., the deoxycorticosterone acetate (DOCA)-salt model [17, 18]. Around the same time, it was concluded that “intracellular renin,” a renin isoform derived from an alternative transcript of the renin gene, lacking the signal peptide and part of the prosegment (and hence being unable to leave the cell) also does not contribute to brain angiotensin generation [19]. In fact, if anything, it suppressed brain RAS activity, although the underlying mechanism remains unknown. Taken together, these data, in combination with the exceptionally low (and often impossible to detect at all) renin mRNA expression of the brain [1•, 20], rule out a role for renin or prorenin in local angiotensin generation at brain tissue sites.

Data on the presence of angiotensinogen in the brain are more convincing. Multiple studies report detectable brain angiotensinogen levels that do not run in parallel with circulating angiotensinogen levels [4, 6]. Yet, generally, brain angiotensinogen levels still at most correspond with a few percent of plasma angiotensinogen levels, in a wide range of species, and thus admixture from blood cannot be entirely ruled out. Importantly, brain angiotensinogen mRNA levels, albeit being several orders of magnitude below those in the liver, are not as excessively low as those of renin [1•]. At this stage, the ultimate proof for local synthesis (showing the presence of angiotensinogen in the brain of animals lacking hepatic angiotensinogen expression) is still awaited. When applying this approach to other organs claimed to synthesize angiotensinogen (kidney and adipose tissue) it turned out that their angiotensin generation depended entirely on hepatic angiotensinogen, implying that local angiotensinogen synthesis in these organs, if occurring at all, has no functional consequence [2, 3, 7]. If angiotensinogen synthesis truly occurs in the brain, a complicating factor remains the absence of renin. This would require non-renin enzymes to cleave angiotensinogen. There is currently no in vivo evidence for this concept.

Angiotensins have been reported in brain tissue in widely varying levels. In some cases, levels (expressed per gram tissue) were even higher than those in the kidney [17, 18]. This is hard to believe given the low angiotensinogen levels, and the absence of renin in the brain. Issues that need to be considered here are the use of very small tissue pieces for angiotensin measurements (often representing selected brain nuclei), the detection limit problems that arise from this approach (inherent to brain research), and the absence of rigorous separation techniques to distinguish true angiotensin from background noise. As an example, measuring an angiotensin (Ang) II level at the detection limit of the assay (often around 2 fmol/sample) in 10 mg brain tissue results in a theoretical tissue level of 200 fmol/g. At the same time, measuring 50 fmol Ang II in 0.5 g renal tissue (i.e., well above the detection limit), would translate to 100 fmol/g. On this basis, it seems that brain Ang II levels are higher in the brain than in the kidney, although in reality, they may be zero.

When employing liquid chromatography-tandem mass spectrometry (LC-MS/MS) to quantify the individual angiotensin metabolites, a highly sensitive method with little or no background noise, we were unable to detect Ang I in brain tissue of spontaneously hypertensive rats (SHR) [1•]. Brain Ang II occurred at levels that were ≈ 25% of the levels in plasma, i.e., they were several orders of magnitude below those in the kidney [21, 22]. Since Ang II type 1 (AT_1_) receptor blockade reduced the brain/plasma Ang II ratio by > 80%, and in view of the absence of Ang I, the most likely origin of brain Ang II is accumulation of circulating Ang II via binding to AT_1_ receptors (explained further later) [21, 23]. Additionally, considering its much smaller size versus renin and angiotensinogen, Ang II may gain access to the brain under conditions where the blood-brain barrier is (partially) disrupted, e.g., in hypertension. In fact, Ang II itself is capable of disrupting this barrier [24, 25]. Once in the brain, Ang II might be converted to metabolites, like Ang III and Ang-(1-7). However, at least in SHR, we were unable to demonstrate these metabolites [1•], and thus whether angiotensin metabolites other than Ang II truly reach meaningful levels in the brain is still uncertain.

## The “Reno-Cerebral Reflex”

**Fig. 2:**
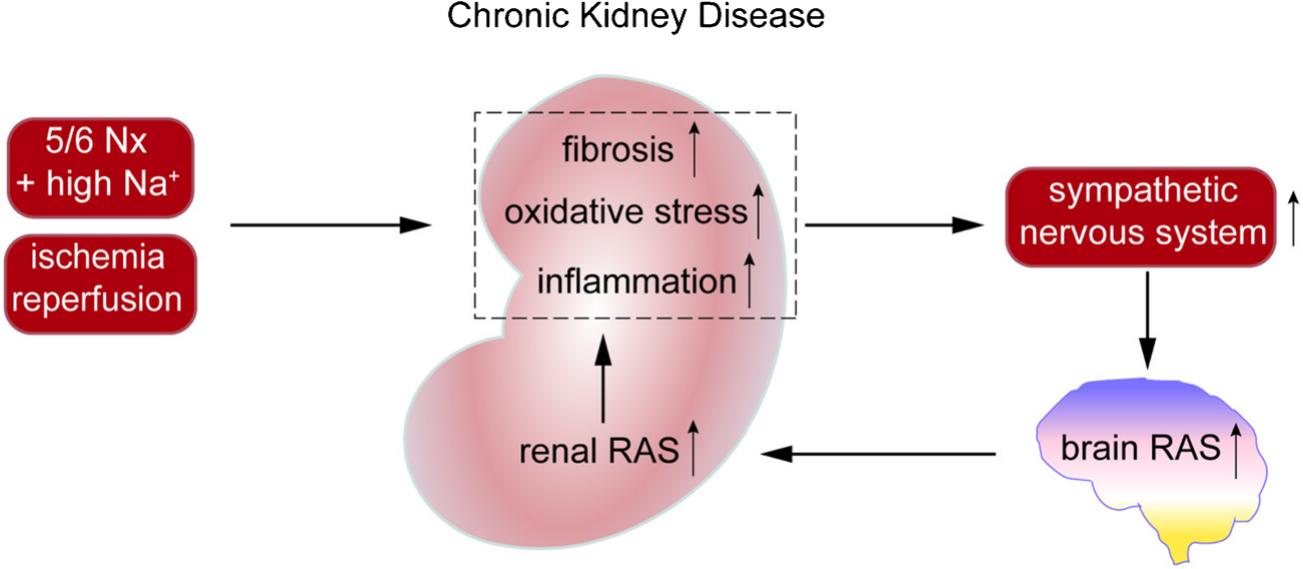
The reno-cerebral reflex. Renal inflammation, fibrosis, and oxidative stress, as occurring in chronic kidney disease models like the 5/6 nephrectomy (Nx) model (in the presence of a high-salt diet) or after renal ischemia + reperfusion, result in sympathetic nervous system activation, which via activation of the brain renin-angiotensin system (RAS) is believed to subsequently upregulate renal angiotensinogen, thereby inducing renal RAS stimulation. A simpler explanation might be that sympathetic activation directly upregulates renal renin synthesis, since this is a well-known consequence of β-adrenergic receptor stimulation, not requiring brain RAS activation

Salt intake promotes progression of chronic kidney disease. Cao et al. have suggested that renal inflammation, as occurring in the 5/6 nephrectomy rat model following exposure to high salt, results in oxidative stress and subsequent activation of the sympathetic nervous system [26•]. Under these conditions, AT_1_ receptors and Ang II (determined semiquantitatively by double-staining immunofluorescence), tyrosine hydroxylase (the rate-limiting enzyme for norepinephrine synthesis), and oxidative stress markers (Nox2 and Nox4) were upregulated in the brain. Remarkably, intracerebroventricular application of losartan or tempol (a reactive oxygen species scavenger) prevented this, as did renal denervation. Moreover, these procedures also prevented the paradoxical upregulation of the renal RAS (reflected by elevated angiotensinogen, ACE and AT_1_ receptor levels) following high salt in this rat model. This eventually led to reduced renal inflammation and fibrosis. Based on these findings, the authors proposed that a there is a “reno-cerebral reflex” resulting in a positive feedback mode, further worsening kidney function (Fig. 2). Similar observations were made in a mouse renal ischemia-reperfusion model under normal salt conditions [27]. Importantly, in both models, the renal RAS upregulation occurred independently of renin, since renin was either unchanged (mouse ischemia-reperfusion model) or severely downregulated (rat 5/6 nephrectomy model during high salt). This is highly unusual, since it is normally renin that allows the up- or downregulation of angiotensin levels [28, 29]. The authors attributed this renal RAS upregulation to the increased renal angiotensinogen expression. Yet, it has already been shown that renal angiotensinogen does not contribute to renal angiotensin generation, neither under normal nor pathological conditions [2, 3]. Furthermore, as an indication of the brain RAS, Cao et al. identified angiotensinogen- and Ang II-positive cells in the brain, making use of anti-angiotensinogen and anti-Ang II antibodies. Given the exceptional low Ang II levels in the brain, Ang II “quantification” by double-staining immunofluorescence should be interpreted with the utmost care, particularly given the inconsistencies obtained with antibodies against RAS components [30]. Why angiotensinogen, a protein which is normally secreted and not stored intracellularly, was observed in brain cells remained unexplained, nor did the authors investigate brain renin. Taken together, although of course the sympathetic connection between brain and kidney is well established (and known to play a vital role in blood pressure regulation and the pathogenesis of hypertension), whether sympathetic activation truly results in significant angiotensin generation at brain tissue sites cannot be concluded from these data. An alternative explanation might be that systemic Ang II, particularly in combination with high salt, via binding to brain AT_1_ receptors outside the blood-brain barrier (e.g., in the subfornical organ or the organum vasculosum laminae terminalis) results in the activation of angiotensinergic projections into brain nuclei within the blood-brain barrier (like the paraventricular nucleus and the rostroventrolateral medulla) [31]. Indeed in rats, subcutaneous infusion of low-dose Ang II alone marginally affected blood pressure, while in combination with high salt, it massively increased blood pressure and upregulated aldosterone [32•]. Central application of either losartan or a mineralocorticoid receptor blocker prevented this. Clearly, these latter data show that protective effects of intracerebroventricular application of an AT_1_ receptor blocker can also be due to interference with systemic Ang II acting in the brain. Similarly, the protective effect of centrally applied mineralocorticoid receptor blockers might relate to blockade of effects of circulating aldosterone in brain nuclei outside the blood-brain barrier, particularly because brain and plasma aldosterone levels were found to correlate closely. In conclusion, before concluding that elevated brain Ang II “positivity” truly reflects activation of the brain RAS based on a reno-cerebral reflex, we need to determine that this is not due to uptake of circulating Ang II into nuclei outside the blood-brain barrier, nor to the disruption of this barrier (as occurring during pathological conditions, including a high-salt diet) causing uptake by nuclei within the blood-brain barrier, like the brain stem and hypothalamus [25, 33]. Here, it is important to realize, as stated above, that Ang II itself is capable of disrupting this barrier. Moreover, central application of an AT_1_ receptor antagonist prevented the central effects of systemic Ang II, and thus effects of centrally applied drugs cannot be taken as evidence for selective interference with brain-derived Ang II. Finally, the sympathetic nervous system is a well-known stimulator of renin release, and thus elevating renal sympathetic nervous activity is likely to activate the renal RAS, yet not necessarily via brain RAS activation.

## Central Aminopeptidase A Inhibition

**Fig. 3:**
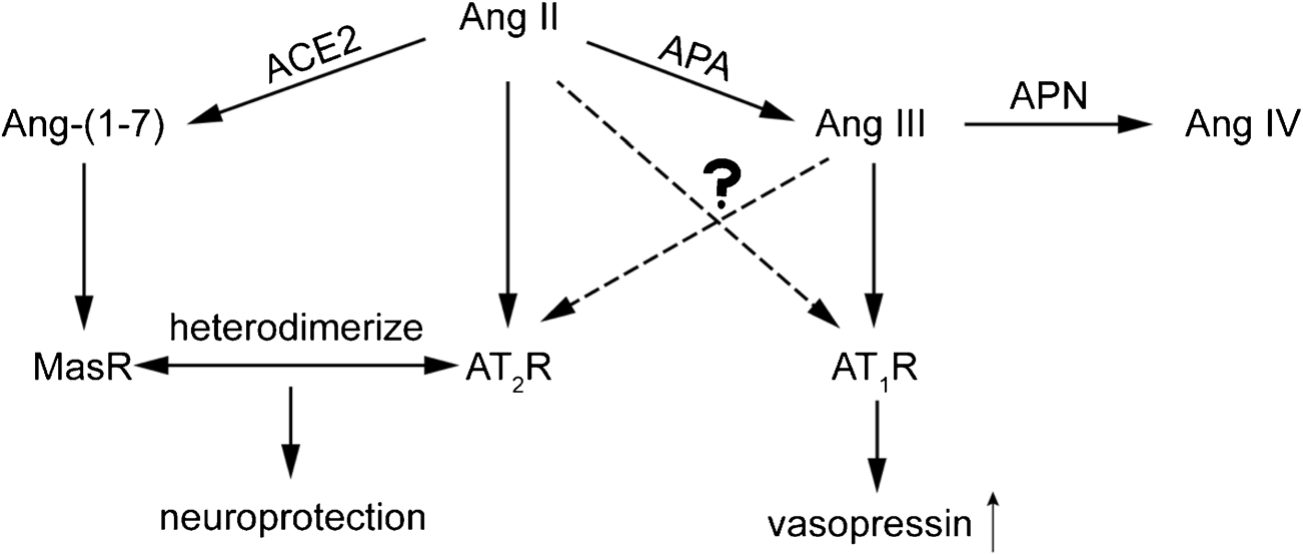
Formation of angiotensin (Ang) III and Ang-(1-7) from Ang II, and the receptors which are believed to be activated by the various angiotensin metabolites. The application of central aminopeptidase A (APA) inhibitors is based on the concept that Ang III is the endogenous activator of AT_1_ receptors, resulting in vasopressin release. Aminopeptidase N (APN) degrades Ang III to the inactive Ang IV. Yet, others argue that Ang II is the preferred agonist of this receptor, while both agonists may also bind to AT_2_ receptors. The latter heterodimerize with Mas receptors, potentially explaining the identical neuroprotective effects observed with AT_2_ and Mas receptor agonists in stroke models

Apart from Ang II, its metabolite Ang III, generated by aminopeptidase A (APA), is an alternative activator of AT receptors. It displays similar affinity for the AT_1_ receptor, and might even be the preferred agonist of the AT_2_ receptor. Assuming that the latter receptor exerts beneficial effects, e.g., coronary vasodilation and diuresis [34–36], blocking APA seems counterintuitive in hypertension. Yet, it has been claimed that in the brain Ang III rather than Ang II is the endogenous AT_1_ receptor agonist, and on the basis of this concept centrally acting APA inhibitors are now being tested in hypertensive patients. This claim relates to the observation that central application of Ang II did not affect vasopressin release (a well-known effect of central AT_1_ receptor activation) when simultaneously blocking APA with EC33, while simultaneous inhibition of aminopeptidase N (APN, the enzyme that degrades Ang III) with PC18 enhanced the effects of Ang II on vasopressin release [37]. Since the Ang II effects were blocked by an AT_1_ receptor antagonist, it was concluded that they involved AT_1_ receptor stimulation by Ang III rather than by Ang II. However, the authors did not quantify brain Ang III in vivo during these procedures, and thus the underlying biochemical evidence for this concept is still lacking. Confusingly, when employing LC-MS/MS we were unable to demonstrate Ang III in the brain [1•], while others observed identical responses to Ang II and Ang III following intracerebroventricular application, even when making use of APA-resistant analogues [38]. An alternative explanation of the findings on APA and APN inhibition during Ang II application should therefore be considered, i.e., the possibility that such inhibition interferes with pathways beyond the RAS. This is not unlikely since these aminopeptidases not only act on multiple substrates, but are also ubiquitously present in- and outside the brain. Nevertheless, an EC33 prodrug, RB150, has now been synthesized which is capable of passing the blood-brain barrier. This drug decreased blood pressure in DOCA-salt rats [39•], but also attenuated cardiac dysfunction after myocardial infarction [40]. As expected, it reduced vasopressin and increased diuresis. A Phase IIa study assessed the blood pressure-lowering effect of a 4-week oral application of RB150 (also known as QGC001 or firibastat) in 34 patients with grade I or II essential hypertension (EudraCT number 2014-003071-37, unpublished results). Although there were no significant effects on blood pressure versus placebo, the drug was found to be safe and did not affect renin. At the recent American Heart Association meeting in Chicago (November 2018), a follow-up trial (NEW-HOPE, NCT03198793, unpublished results) in a much larger hypertensive, overweight subject population of multiple ethnic origin (*n* = 250) reported antihypertensive effects of the drug (250 mg, 500 mg, or 500 mg + 25 mg hydrochlorothiazide) over an 8-week treatment period. A detailed analysis of its dose-dependency and the effect of adding hydrochlorothiazide was not provided, and the trial did not include a placebo arm. Taken together, APA inhibition may well be a promising novel treatment strategy in patients displaying low systemic RAS activity (like in DOCA-salt-treated rats) but whether this truly involves suppression of brain Ang III remains to be proven (Fig. 3).

## AT_2_ and Mas Receptor Agonism in the Brain

AT_1_ receptor blockers have often been suggested to offer cerebrovascular protection, in contrast to ACE inhibitors, although clinical evidence for this concept is still missing [41]. The underlying mechanism of this concept would be that only AT_1_ receptor blockers allow central AT_2_ receptor agonism. Possibly, AT_2_ receptor stimulation by endogenous Ang II might already exert protective effects. Indeed, the neurological deficit after middle cerebral artery occlusion was greater in AT_2_ receptor knockout mice, and without AT_2_ receptors, the beneficial effects of AT_1_ receptor blockade were diminished [42]. Now that AT_2_ receptor agonists like C21 are available, the next step is to evaluate these drugs in stroke models, like the middle cerebral artery occlusion model and the endothelin-1-induced ischemic stroke model. Indeed, in both models, C21 exerted cerebroprotection, not only when applied intracerebroventricularly, but also when applied systemically [43, 44]. To explain the efficacy of the latter approach, considering that C21 cannot pass the blood-brain barrier, it has to be assumed that C21 enters the brain under conditions where this barrier has been disturbed, like in the above models. An exciting novel approach is to administer C21 via the nose-to-brain route to bypass the blood-brain barrier [45•]. When applying C21 via this route at 1.5 h after stroke, it reduced infarct size and improved neurological scores. Furthermore, angiotensin-(1-7) (Ang-(1-7)), generated from Ang II by ACE2, also offers neuroprotection, both when applied centrally and orally, as did the putative ACE2 activator diminazene [46]. Ang-(1-7) is believed to act via Mas receptors. One possible explanation for the identical beneficial effects of AT_2_ and Mas receptor stimulation is that both receptors co-localize and are functionally interdependent [47]. Importantly, none of these approaches supports actual angiotensin synthesis in the brain allowing AT_2_/Mas receptor activation by endogenous angiotensins. A further complicating factor is that both C21 and diminazene exert AT_2_ receptor- and ACE2-independent effects, respectively [48, 49], while Ang-(1-7) was recently reported not to act as Mas agonist at all [50]. Clearly, these observations remain controversial, and even if they can be taken as evidence for AT_2_/Mas receptor activation, they do not automatically imply that these receptors are normally seen by brain-derived endogenous agonists. In fact, their stimulation may depend on breakdown of the blood-brain barrier (Fig. 1), allowing circulating angiotensins access to brain receptors [42].

## Conclusion

Convincing evidence that angiotensin synthesis occurs independently at brain tissue sites is lacking. Renin is absent and brain angiotensin levels are exceptionally low as compared to other organs. In fact, they may well represent binding of circulating Ang II to brain AT_1_ receptors in brain nuclei outside the blood-brain barrier. To investigate whether brain-originating angiotensinogen, if existing, contributes to brain angiotensin synthesis, experiments need to be performed under conditions where hepatic angiotensinogen synthesis is silenced, preferably in a model where brain angiotensin is assumed to play an important role, like the DOCA-salt rat. Before concluding that novel therapies aimed at APA, APN, ACE2, AT_2_, and Mas receptors interfere with the brain RAS, we not only need to exclude non-specific effects of the applied drugs, but also show that they truly affect brain angiotensin levels, and that this explains their effects. In other words, it would help to demonstrate brain-selective Ang III suppression during RB150 treatment, and Ang-(1–7) upregulation after diminazene. Here, the application of a highly sensitive method with little or no background noise like LC-MS/MS is essential. Yet, given the fact that APA, APN, and ACE2 have multiple other substrates, one simultaneously needs to rule out that effects are seen due to interference with these alternative substrates.
